# Nanotechnology Facilitated Cultured Neuronal Network and Its Applications

**DOI:** 10.3390/ijms22115552

**Published:** 2021-05-24

**Authors:** Satnam Singh, Sachin Mishra, Song Juha, Manojit Pramanik, Parasuraman Padmanabhan, Balázs Gulyás

**Affiliations:** 1 Biomanufacturing Technology, Bioprocessing Technology Institute (BTI), Agency for Science, Technology and Research (A*STAR), 20 Biopolis Way, Singapore 138668, Singapore; Satnam_Singh@bti.a-star.edu.sg; 2 Cognitive Neuroimaging Centre, Nanyang Technological University, 59 Nanyang Drive, Singapore 636921, Singapore; sachin.mishra@ntu.edu.sg; 3 Lee Kong Chian School of Medicine, Nanyang Technological University, 59 Nanyang Drive, Singapore 636921, Singapore; 4 School of Chemical and Biomedical Engineering, Nanyang Technological University, 62 Nanyang Drive, Singapore 637459, Singapore; songjuha@ntu.edu.sg (S.J.); manojit@ntu.edu.sg (M.P.); 5 Department of Clinical Neuroscience, Karolinska Institute, S-171 76 Stockholm, Sweden

**Keywords:** nanotechnology, neuronal network, neural stem cells, nanopatterning, nanoelectrode, neuronal signal recording

## Abstract

The development of a biomimetic neuronal network from neural cells is a big challenge for researchers. Recent advances in nanotechnology, on the other hand, have enabled unprecedented tools and techniques for guiding and directing neural stem cell proliferation and differentiation in vitro to construct an in vivo-like neuronal network. Nanotechnology allows control over neural stem cells by means of scaffolds that guide neurons to reform synaptic networks in suitable directions in 3D architecture, surface modification/nanopatterning to decide cell fate and stimulate/record signals from neurons to find out the relationships between neuronal circuit connectivity and their pathophysiological functions. Overall, nanotechnology-mediated methods facilitate precise physiochemical controls essential to develop tools appropriate for applications in neuroscience. This review emphasizes the newest applications of nanotechnology for examining central nervous system (CNS) roles and, therefore, provides an insight into how these technologies can be tested in vitro before being used in preclinical and clinical research and their potential role in regenerative medicine and tissue engineering.

## 1. Introduction

Understanding the fundamental biology of neural tissue growth and synapse formation assists to define overall functions performed by these tissues [1]. Owing to the complexity of the nervous system, to study neural activities such as synaptogenesis and axonal pathfinding, it is very crucial to isolate neuronal cells from their tissue niche and cultivate them in vitro [2]. Moreover, for the controlled growth of neural stem cells (NSCs) and their differentiation into other cell types such as neurons and glial cells expressing oligodendrocyte and astrocyte lineage markers, an accurate balance of microenvironment factors is required [3,4]. Moreover, the complex neuronal network at different levels, from a minor network of several neurons to a huge assembly of thousands of cells, enables the neuronal network to perform computations with astonishing reliability in a very short timescale. Consequently, the goal to design and develop a well-controlled neuronal circuit outside the human brain has encouraged scientists to discover new nanotechnology-assisted techniques to construct biomimetic neuronal networks in vitro [2]. These studies aim to give new insight into the development of in vitro neuronal testbeds for validation of various pathogenic mechanisms or new drugs prior to their use in preclinical and clinical applications for the treatment of neuronal diseases and, eventually, their potential roles in regenerative medicine and tissue engineering.

**Figure 1:**
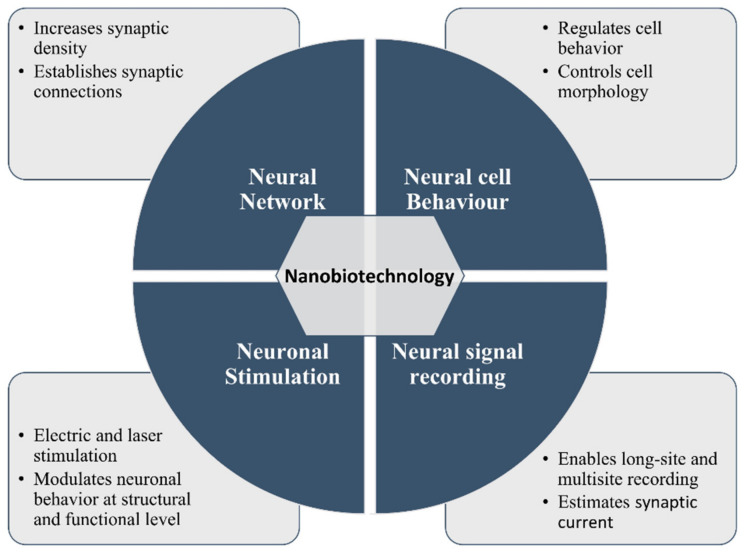
Illustration of nano-biotechnological approaches for neuroscience research.

Over the last few years, nanotechnology has been emerging very fast for its applications in biological and biomedical sciences. This nanotechnology is generally defined as the manipulation of functional structures that have at least one dimension size from 1 nm to 100 nm [5]. However, there are different critical steps involved in designing, creating, and optimizing nanostructures through nanofabrication methods, such as bottom-up synthesis of nanopatterned surfaces, in addition to the characterization and evaluation of nanoscale matters in conjunction with nano- or microinstrumentation. In this paper, we have reviewed recent advances on different nanomaterials for their use in NSCs’ growth and differentiation in vitro. We have highlighted four key nanotechnological aspects in artificial neuronal networks: (i) nanomaterials for neuronal network establishment, (ii) nanostructural design and fabrication for cell morphology and fate, (iii) nanotechnology-assisted neuronal stimulation, and (iv) nanodevices for neuronal signal recording (Figure 1).

## 2. Nanotechnology for Neuronal Research

### 2.1. Nanomaterials for Neuronal Network Establishment

Many efforts have been made to uncover the underlying molecular and cellular mechanisms associated with the formation of neuronal circuits. Scientists are working on developing biomimetic and biocompatible support to control NSCs’ growth and differentiation into ordered neuronal networks [6,7,8]. The growth supports are engineered to form 3D architecture for guiding primary brain cells to form in vitro neural circuits. Various types of surface chemistries of support materials regulate cell adhesion, spreading, elongation, shape, and finally, cell fate [9,10]. This allows neurons to reform synaptic networks in suitable directions in 3D architecture. The presence of neuroglia in a homeostatic environment in a 3D neuronal network might offer a critical model of the central nervous system (CNS) with multilevel incorporation of signals in health and disease [6,7,8]. For instance, primary neurons grew on 3D surfaces, such as combined silica beads, and the bottom layer of these neurons was additionally interfaced to a 2D Microelectrode Array (MEA) [11]. In most cases, 3D electrospun polymers or hydrogels have been used for the development of genuine in vitro tissue models, and they are likely to be degraded by astrocytes present along with neurons in a short time, whereas robustness in time is a requisite to study in vitro mechanisms of CNS growth or disease [12].

For the modulation of the cell morphology by means of nanotechnology, the synthetic nanosized materials offer inimitable surface properties that can influence cell behavior to establish a neuronal network [13]. The imposition of specific geometries on biocompatible support surface leads to change its surface–volume ratio, wetting behaviour and roughness, which could resemble surface design close to the molecular/cellular scale [14,15]. The substrate topographies influence the directional growth of neuronal cells and the degree of adhesion between cells and surface by physical confinement and chemically functionalized surface, which are significant to enhance or inhibit cell process growth and importantly to identify the appropriate targets for establishing neural synaptic connections [16].

By designing the nanoscale topography on the cell culture substrate (growth surface), the specific role of physical guidance in the formation of neuronal circuits can be understood [17]. The ability of neural cells to respond to nanostructured topographies/patterns features has already been described in the literature [16,17]. The aforementioned properties of nano/micron level materials have shown that neurons and astrocytes could be cultured on the nanogrooved surface with various depths for different purposes [16]. Concerning this, rat hippocampal neurons were cultured on poly-L-lysine-coated silicon surfaces comprising of pillars (~1 μm in height) with varying width and spacing. This platform has resulted in a significantly longer neurite growth with 2 μm pillar widths and minimum inter-pillar gaps. One of the possible reasons behind this result could be the resembling feature of pillars with topographies exhibited by ECM; for instance, the basement membrane of the corneal epithelium has topographies such as pores, fibrils, and pillars made up rigid structural proteins [18]. Additionally, a pillar-like pattern (ridge–groove–ridge) directs the growth by providing a surface for neurite attachment (contact guidance) and influences the alignment of extracellular and intracellular proteins on the nano/micropatterned surface [19]. These strategies would facilitate the formation of the synapse and will aid in the establishment of a neural network. A study by Cellot (in 2017) compared cultured neurons on Carbon Nanotubes (CNT) with a control surface and showed CNT doubled the probability of synapse development. The reason behind the robust coupling probability was an increased synaptic density, which enhanced the GABAergic synaptic contact in cultured neurons on CNT layers. This significant increment of neuronal network connectivity on CNTs led to an upsurge in the Postsynaptic Currents (PSCs) of the neurons [20,21,22].

**Figure 2:**
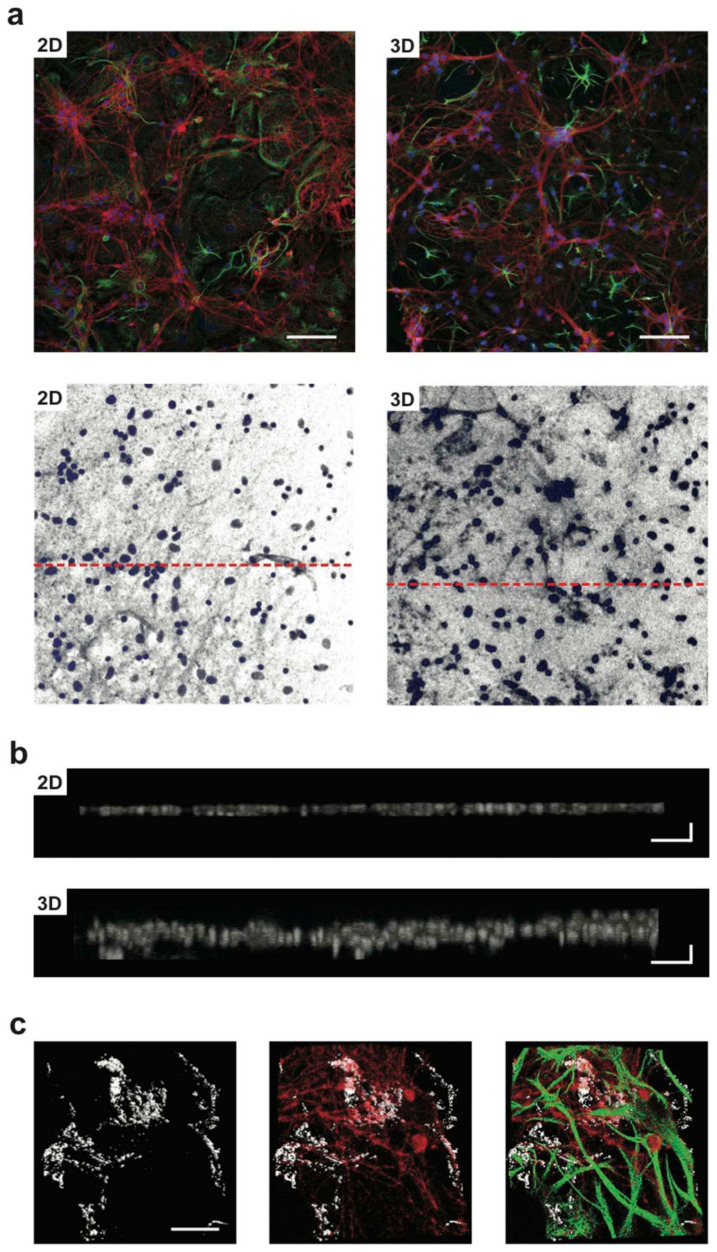
The development of primary neurons in 2D- or 3D-PDMS scaffolds. In (**a**) (top row), confocal micrographs show hippocampal cultures grown (9 DIV) on 2D-PDMS (left) and 3D-PDMS (right) immune-stained for β-tubulin III (in red), GFAP (green), and DAPI (blue). Scale bar: 100 mm. In the bottom row’s images, only the DAPI channel is selected to highlight the nuclei under the two culturing conditions (same visual fields as in (**a**) top); the dashed red lines represent the regions for which the z profile reconstructions are performed in (**b**) note the increased thickness of DAPI signal in the 3DPDMS. Scale bar: 100 × 10 μm. In (**c**), a confocal reconstruction of a 3D-MWCNT scaffold (left; in grey carbon nanotubes are visualized by confocal under reflection mode acquisition, allowing to visualize the scaffold structure); confocal reconstruction of neurons (in red; middle) grown suspended within a pore and glia cells (in green; merged in the right panel) acting as a support. Note the complex growth of neuronal and glial processes exposed to the third dimension. Scale bar: 50 μm. Adapted with permission from Ref. [6].

Moreover, the improvement in the neural synaptic connections on 2D and 3D porous scaffolds made up of polydimethylsiloxane (PDMS) (named 2D-PDMS and 3D-PDMS, respectively) was seen in nanotechnology applications. The micrometric cavities present in the porous PDMS scaffolds were exposed to the Multi-Walled Carbon Nanotubes (MWCNTs). This permits the fabrication of scaffolds with holes layered by an irregular MWCNT carpet of around 100 nm thickness. The resulting 2D and 3D scaffolds with MWCNT boost synaptogenesis to form a synaptic network of hippocampal cells and have been confirmed using immunofluorescence staining, confocal microscopy, and Ca^2+^ imaging techniques (Figure 2) [6].

**Figure 3:**
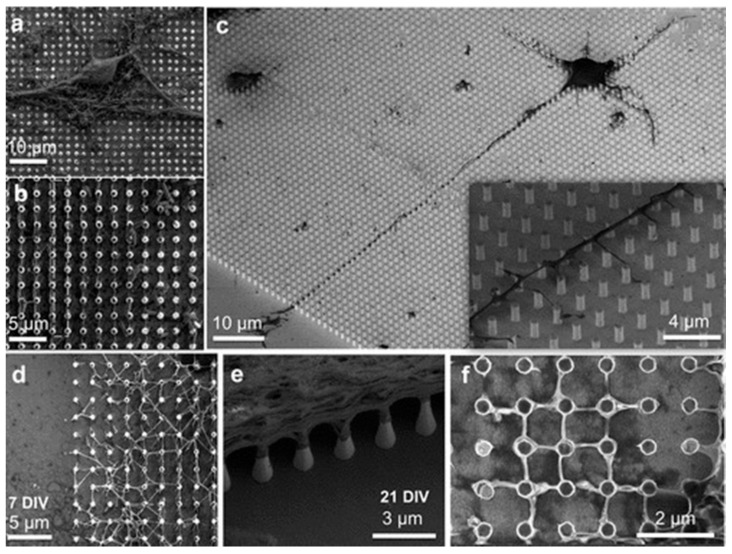
SEM images of the growth of neurons and neurites on nanowire arrays. (**a**) A cell body from hippocampal culture on the nanowire array, top view, after 20 DIV. (**b**) Another area on the same substrate as in panel (**a**), where neurites can be seen to grow along the nanowires, top view. (**c**) A neuron from cortical culture after 2 DIV. The inset depicts a closer view around the axon. (**d**,**e**) Cellular network on the edge of the nanowire array after 7 and 21 DIV, respectively. (**f**) Neurite growth on an area of nanowires after 5 DIV showing anchoring and secondary branching of neurites at the nanowires. Adapted with permission from Ref. [17]. Copyright (2017) American Chemical Society.

Semiconductors, at a nanoscale level similar to nanowires, also have been used for their potential incorporation within electronic circuits for stimulating and recording cellular activities. Recently, for the first time, Indium Phosphide (InP) nanowire scaffolds mesh of 200 × 200 μm (100–800 nm dia and height around 2 μm) was used to support neuronal development and the formation of a well-connected neuronal network. InP nanowire is a direct band gap semiconductor unlike gallium phosphide (GaP) and silicon (Si) and provides a superior optoelectronic interface to stimulate neurons [17,23]. The first demonstration of biocompatibility of InP-based optoelectronic substrates has been shown to serve as physical cues for in vitro neural cell growth and their alignment to form a network with neurons. The result showed the growth of cortical and hippocampal neurons in a controlled manner from 2 days in vitro (DIV) to 21 DIV to form the interconnection between neurites. The increased density of neurites was seen at the region of nanowires to form a neuronal network, and this network terminates at the boundary between nanowires and also at smooth InP surface (Figure 3) [17,23,24].

The role of substrate roughness (S_a_) is significant in neuronal network establishment and information processing, as found using fluorescent multi-calcium imaging and computer simulation methods. According to a study, neural cells seeded for 11 days onto corrugated surfaces (S_a_ > 22 nm) show small-world attributes, which enhanced exchanged information by 4-fold as compared to neural cells on a flat surface (S_a_ < 10 nm), which show uniform distribution over the surface with no clustering effects [25].

Furthermore, Superhydrophobic (SH) surfaces fabricated of nanopatterned silicon (cylindrical pillars of 10 μm diameter and height, organized in hexagonal mesh with a periodicity of 30 μm) with deposition of 5 nm thick layer of Teflon-like (C_4_F_8_) polymer can enhance cell survival, growth, and differentiation of primary hippocampal neurons, seeded on them. Considering the necessity of a polycationic nature of the surface for the cell adhesion to the substrate, the standard poly-D-lysine (PDL) was coated on the surface, much concentrated on the top and base of the pillars and very less at the lateral surface and was imaged by confocal microscopy. The sidewalls of the pillars were either smooth or nanopatterned of grooves to influence cell growth and network establishment. Comparing the growth of neuronal cells to form a network, a rough nanopatterned surface better supports the 3D network of cells as compared to a smooth surface.

In the early stage of cell seeding, neuron growth was found on the top of the pillars, which was followed by their strong contact with the sidewall of roughed pillars and confirmed with concentrated Neuronal Cell Adhesion Molecule (NCAM) in these regions, which indicates strong adhesion of neurons to rough surfaces. Unlike smooth pillars, this would lead to keep cell bodies in the suspended 3D network. On the contrary, weaker contact of cell processes with smooth pillars could not support the 3D network formation, leading to a 2D network where neurons and processes are restricted to the bottom part of pillars and therefore lay at the pillar’s bottom [14].

Hence, the aforementioned properties of nanomaterial are very valuable for the directional growth of neurons to form synaptic connections and develop appropriate neural networks, which can be applied for regenerative medicine research.

### 2.2. Nanostructural Design and Fabrication for Programming Cell Behavior

Cell behavior and morphology are dependent on the physical microenvironment. As stem cells are sensitive to surface structure, scientists have modified the surface chemistry/pattern of the substrate to control cell adhesion, shape, elongation, spreading and differentiation [9,26]. The surface patterning of the substrate by either micro or nanoengineering techniques also influences stem cell differentiation. These micro/nanopatterning can be conducted by using hard lithography technique (photolithography) or soft lithography technique (microcontact printing) [27]. The most common shapes, such as squares, strips, circles, grooves, triangles, and grids, can be developed by the above-mentioned techniques [27]. Moreover, substrate curvatures such as convex or concave might also influence neuronal polarity, ion channels, differentiation etc. [28].

Concerning this, nanocomposites template comprising of graphene oxide (GO) and conducting polymer poly(3,4-ethylenedioxythiophene) (PEDOT) has been used for the differentiation of NSCs [29]. Graphene nanogrids (crossed graphene nanoribbons) with nanoribbons dimensions (length ~10 mm, width ~50–200 nm, and thickness 1 nm) were also used to induce higher neural differentiation of hNSCs into neurons [30]. The coating of nanodiamond (ND) of different sources, sizes, surface chemistries, and deposition methods on glass cover slips showed promising results for in vitro study of the murine hippocampal neuronal network without means of other biomolecules for adhesion [31]. However, recently in 2017, surface-functionalized ND with oxygen (O-ND) and hydrogen (H-ND) exhibited different results in terms of cell adhesion and cell count. O-ND coated Poly-L-lysine and Laminin (PL + LN) showed high cell adhesion and count, unlike Tissue Culture Polystyrene (TCPS) alone. Moreover, hNSCs culture on both glass and H-NDs also resulted in lower cell adhesion and cell counts. The possible reason for this result could be the contact angle, which was much lower for O-NDs than others which leads to enhanced hydrophilicity [32].

**Figure 4:**
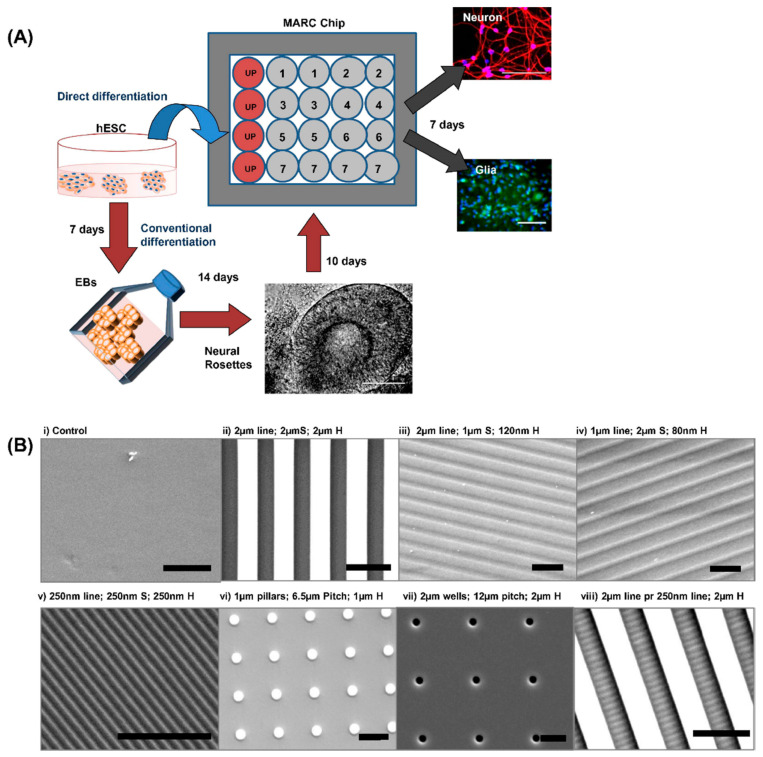
(**A**) A schematic of human embryonic stem cell (hESC) neural differentiation into neurons and glial cells on the multi-architecture chip (MARC) with minimal neuronal supplements by the direct or conventional method. In “direct differentiation” (blue arrow), the hESCs were seeded directly on the MARC and analyzed for neural markers after 7 days. In “conventional methods” (red arrows) of differentiation, hESCs were grown as embryoid bodies and neurospheres before seeding onto the MARC. On the MARC, each pattern is represented by a circle and has a duplicate. The red circles represent the unpatterned control surface. Scale bar: 100 μm. (**B**) (**i**–**viii**) Patterns are replicated with high fidelity on polydimethylsiloxane (PDMS) as verified by scanning electron microscopy (SEM) images. The geometries are transferred onto PDMS from the MARC master molds by soft lithography. (**i**) Unpatterned PDMS control, (**ii**) 2 μm grating with 2 μm spacing and 2 μm height, (**iii**) 2 μm grating with 1 μm spacing and 120 nm height, (**iv**) 1 μm grating with 2 μm spacing and 80 nm height, (**v**) 250 nm grating with 250 nm spacing and 250 nm height, (**vi**) 1 μm pillar with 6.5 μm pitch and 1 μm height, (**vii**) 2 μm wells with 12 μm pitch and 2 μm height, (**viii**) hierarchical structure having 250 nm gratings with 250 nm space perpendicular to the 2 μm grating. The spacing and height between the 2 μm gratings is also 2 μm. Scale bars: 5 μm. Abbreviations: “S” refers to the spacing between the gratings, “H” refers to the height of the topography and “perpendicular to” is abbreviated as “pr.” Adapted with permission from Ref. [33].

Moreover, the various nanotopographies on a chip called a multi-architectural chip (MARC) had made it possible to screen more than one topography at a time and formulating their complex structure on a chip. This chip can work as high-throughput screening of different topographies with different properties, which can possibly maximize the neuronal differentiation efficiency from pluripotent stem cells. MARC comprised both anisotropic (such as gratings) and isotropic patterns (such as pillars and wells) along with hierarchical structures. The human embryonic stem cells (hESCs) seeded onto poly-L-ornithine (PLO) and laminin-coated MARC and immunofluorescence staining after 7 days showed that anisotropic pattern enhanced neuronal differentiation of hESCs, whereas isotropic patterns enhanced glial differentiation of hESCs (Figure 4) [33].

Laser exposure and electromagnetic field also influence the growth and differentiation of the neural cells when cells encounter nanomodified gold. The exposure of 780 nm laser with lower power on NG108-15 mouse neuroblastoma cultured with gold nanorods (NRs) showed increased neurites number per neuron and increased average length of neurites. This study data showed that laser exposure did not produce any permanent cell damage. However, the effect of laser exposure on the average length of neurites was significant and positively correlated with laser power. With the irradiation of 7.5 W/cm^2^, the greatest length of neurite was increased on average by almost 36% higher when compared to non-irradiated cells. The reason behind this phenomenon was hypothesized that the transient heat produced by excitation of localized surface plasmon resonance in NRs might generate extra Reactive Oxygen Species (ROS). This increased level of ROS can increase the cell metabolic activity by activating the transcriptional factors [34].

**Figure 5:**
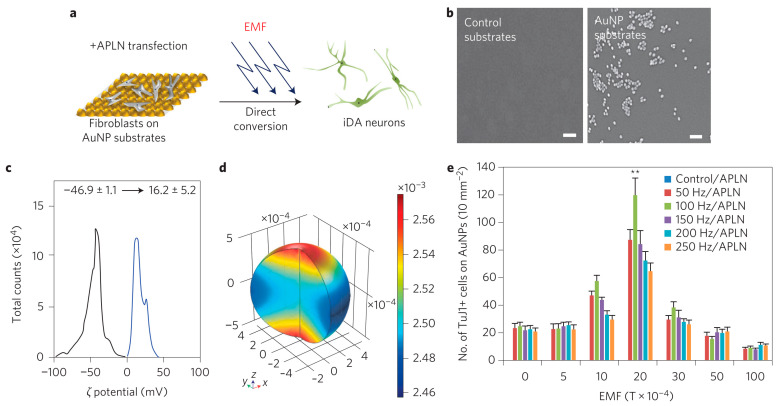
Schematic illustrations showing the process for direct lineage reprogramming into iDA neurons using EMF-induced AuNP magnetization. (**a**) Mouse fibroblasts transiently transfected with APLN were plated on the AuNP substrate and exposed to a specific frequency and intensity of EMF. (**b**) SEM images of the control substrate and the RGD−AuNP-coated substrate. Scale bars, 100 nm. (**c**) The surface charge of citrate−AuNPs (black) and RGD−AuNPs (blue) was determined by zeta-potential measurement. The zeta potential of the AuNPs shifted from −46.9 ± 1.1 mV to 16.2 ± 5.2 mV on ligand exchange. (**d**) The calculation of the magnetic flux spatial distribution on the surface of AuNPs during EMF exposure (100 Hz and 2 × 10^−3^ T). (**e**) The number of TuJ1+ cells generated on magnetized AuNPs under different intensities and frequencies of EMF exposure. Data are represented as mean ± s.e.m. (*n* = 5). ** *p* < 0.01, one way ANOVA. Adapted with permission from Ref. [35].

Recently, Yoo et al. (2017) synthesized electromagnetized gold nanoparticles (AuNPs), which facilitated somatic cell lineage reprogramming into induced dopaminergic (iDA) neurons when given specific electromagnetic field (EMF) conditions (Figure 5) [35]. Cha et al. (2017) created polystyrene cell culture dishes with omnidirectional nanopore arrayed surface (ONAS) with 200 nm diameter, 500 nm center-to-center distance, and 500 nm depth. The proliferation of rat NSCs on ONAS showed more proliferated cells and reduced differentiation in the presence of mitogens as compared to flat surfaces, facilitating NSCs’ proliferation. In the case of ONAS, interestingly, proliferated cells formed neurosphere and migrated out, whereas, on flat surfaces, proliferated cells migrated individually [36]. Some scientists also compared Cerium oxide nanoparticles (CeO_2_ NPs) and Samarium (Sm) doped CeO_2_ NPs (Sm-CeO_2_). They concluded that CeO_2_ NPs suppress the specific *β*3-tubulin expression (neuronal differentiation marker), which leads to the inhibition of NSCs differentiation due to their antioxidant properties, whereas, it was not the same in the case of Sm-CeO_2_ [37].

### 2.3. Nanotechnology-Assisted Neuronal Stimulation

Electric stimulation of various cells has been widely used to treat several conditions related to musculoskeletal and neurological disorders and provided a significant impact on laboratory research. It balances the electric signal propagation by compensating for the altered electric activity of cells and improve their growth and tissue generation properties. Owing to the intrinsic electroactivity properties of nerve cells, scaffolds with conductive properties and the ability to deliver electric stimulation have gained interest for their application in neuroscience. Numerous ways of invasive and non-invasive stimulation have been reported and practiced to precisely confine the activation of specific nervous structures. The exclusive mechanical, electric and biological properties of different nanoparticles make them a potential candidate for providing a neural interface to decide neural cell fate (i.e., viability, migration, division, and differentiation) and neural network formation [38,39].

Nowadays, piezoelectric materials are widely being used in biomedical research due to their fascinating property of generating electric fields upon applying mechanical stress, called the “direct piezoelectric effect.” Many in vitro studies have shown that different cell types behave differently when incubated with piezoelectric substrates/scaffolds.

Ciofani et al. (2010) reported the use of Boron Nitride Nanotubes (BNNTs) (which is analogous to CNTs) and ultrasound to stimulate neuronal-like cells in culture. Despite the structural similarity of BNNTs with CNTs, BNNTs show superior chemical, mechanical and electrical properties. They proposed a unique way to stimulate the cells in vitro, based on the piezoelectric properties of BNNTs without the usage of electrodes in culture. BNNTs of length 200–600 nm and diameter of 50 nm, incubated with the neuronal-like PC12 cells and ultrasounds were used to deliver mechanical stress to BNNTs. This led to the polarization of nanotubes due to the piezoelectric properties of BNNTs and delivered electrical stimulus to the cells. PC12 cells stimulated with this innovative technology showed a 30% increment in neurite sprout after 9 days of treatment.

As suggested by Ciofani et al., this concept model can also be implied in life science to electrically stimulate the cells when required [38]. Similarly, tetragonal barium titanate nanoparticles (BTNPs) with ultrasound treatment are used to stimulate SH-SY5Y cells to provoke a notable cellular response by activating high amplitude Ca^2+^ transients, known as Ca^2+^, whereas only ultrasound stimulation without BTNPs could induce Ca^2+^ transients of low amplitude. Furthermore, these Ca^2+^ waves are known to propagate intercellularly through gap junctions on adjacent neurons. These waves are important in the establishment of a neural network, especially by controlling the neurite outgrowth [40,41].

Moreover, neuronal stimulation mediated by laser has opened another potential field of research. In this technique, a laser pulse is used to stimulate cells instead of an electric field, which provides superior spatiotemporal resolution by avoiding the electronic crosstalk. Recently, a similar study by Johannsmeier et al. (2018) demonstrated gold nanoparticle facilitated laser stimulation to excite the cells and studied calcium (Ca^2+^) response in murine Neuro-2A (N2A) cell line in primary mouse cortical neurons. Gold nanoparticles (AuNPs) excite the cells by transferring energy from a laser pulse to the cell membrane. By visualizing the lipid peroxidation and calcium flux in cells by fluorescent dye, they hypothesized that when AuNPs are irradiated at their plasmon resonance frequency, the release of Ca^2+^ from the endoplasmic reticulum is triggered [42]. Ca^2+^ acts as universal messengers and plays a crucial role in numerous signaling pathways and cellular stress response. Furthermore, Ca^2+^ is a source of cell stress and tracking the calcium flux of AuNPs-laser-treated cells can reveal information on different aspects of Ca^2+^ role and health [43,44]. Generally, NP’s resonance frequency is tuned by altering their composition, aspect ratio and shape [45]. By translating this mechanism into in vivo applications, we can develop a safe neural implant to provide a healthy biological interface and reduce adverse effects by providing required stress by a laser pulse.

**Figure 6:**
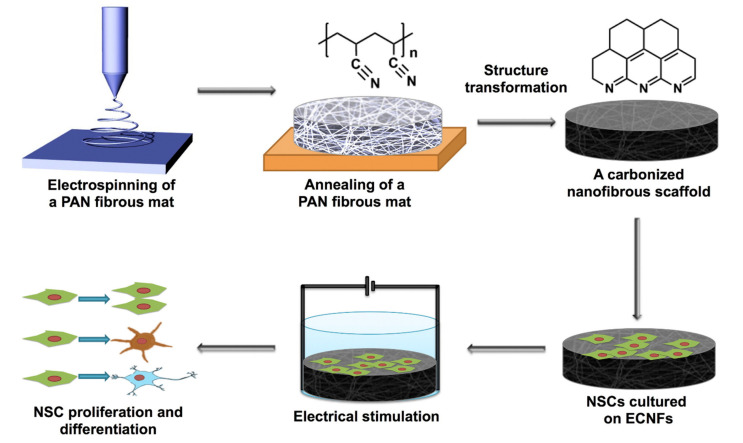
A schematic illustration of ECNF scaffold fabrication and electrical stimulation of NSCs on the scaffold. Adapted with permission from Ref. [50].

Carbon-based nanomaterials, such as carbon nanofibers (CNFs), carbon nanotubes (CNTs), and graphene, have shown their potential roles in neuroscience due to their mechanical, electrical and biological properties [46,47]. Researchers have demonstrated that CNTs can modulate neuronal behavior at structural and functional levels (such as neurite elongation and synaptic efficacy, respectively) [47,48,49]. By integrating electric stimulation with electrospun carbon nanofibers (ECNFs) scaffold, Wei Zhu et al. promoted the NSCs’ proliferation, differentiation, and maturation, associated with upregulation of specific genes. The above-research findings showed the potential of this technique for use in neural tissue regeneration (Figure 6) [50]. The above-discussed research provides knowledge about the interaction between different nanostructures and neuronal cells, especially how cells cultured on nanostructures can be stimulated for different purposes. Future work should focus on translating this technology into biological and clinical practices.

### 2.4. Nanodevices for Neuronal Signal Recording

**Figure 7:**
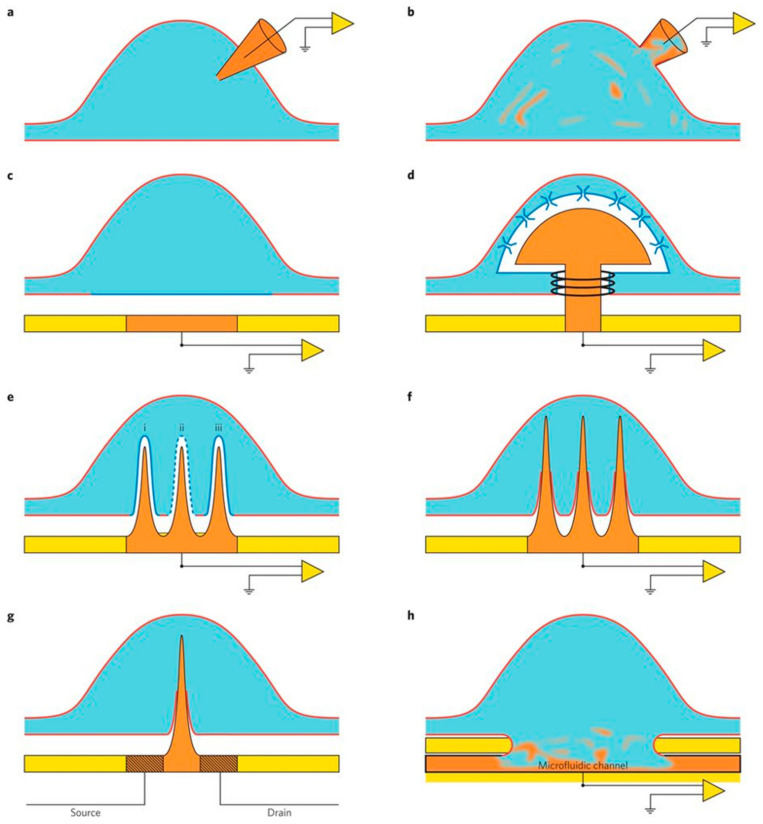
Different forms of the electrode/neuron and cardiomyocytes interface configuration. (**a**) A sharp glass intracellular microelectrode. (**b**) Whole-cell patch-electrode configuration. The ‘mixing’ of orange and blue schematically illustrates the perfusion of the cytosol by the electrode content. (**c**) A neuron cultured on a substrate-integrated planar extracellular electrode. Note the cleft (white) separating the junctional membrane and the electrode. (**d**) A neuron engulfing a gold mushroom-shaped protruding microelectrode. Note actin rings surrounding the mushrooms stalk, stabilizing the configuration. (**e**) Nanopillar electrodes extending into a cultured cardiomyocyte but do not penetrate the plasma membrane (**i**). After the application of an electroporating pulse, (**ii**) the nanopillar gains access to the cytoplasm. The electroporation is transient, and the junctional membrane resistance recovers to control level within minutes (**iii**). (**f**) An array of nanopillars that penetrate the plasma membrane forming direct physical contact with the cytosol. (**g**) A nanopillar that serves as the gate for a nanoFET penetrates the cell’s membrane. (**h**) Patch clamping of cultured neurons. The mixing of the ionic solution of the microfluidic system with the cytosol is depicted. For more details, see reference. Adapted with permission from Ref. [52].

At present, the primary aim of neuroscience is to discover the relationships between neuronal circuits and their pathophysiological functions. To do so, various technologies have been developed using micro and nanotechnology for neuron signal recording in in vitro, such as patch-clamp array for intracellular recording and substrate-integrated MEAs for extracellular recording. Both the techniques have some limitations; for instance, the patch-clamping technique permits high-precision intracellular recording but is not appropriate for the network-level investigations whereas, MEAs enable extracellular neuronal network recoding precisely and are not very sensitive for intracellular recording [51,52]. The perfect device with a multiunit system should offer electrophysiological parameters information from individual neurons, including Action Potential (APs), subthreshold Inhibitory Postsynaptic Potentials (IPSPs) and subthreshold Excitatory Postsynaptic Potentials (EPSPs), and subthreshold membrane oscillations. By combining both nano and microtechnology, scientists have developed a device that enables simultaneous, long-site, and multisite recording (Figure 7) [52].

Patch-clamp electrodes and glass electrodes are known for intracellular recording by producing seal resistance (R_seal_) with the plasma membrane when they penetrate it. Robinson et al. developed vertical nanowire electrode arrays (VNEAs) from silicon-on-insulator, and each NW in the array comprises of doped silicon core encapsulated by silicon dioxide and sputter-deposited metal tip Ti/Au. The metal tip and silicon core were used for providing internal access to the cell, and the glass shell prevents current leakage and tightly seals the cell membrane. The developed VNEAs had 16 stimulation/recording pads and lied into 3 × 3 arrays of nine silicon NW (150 nm in diameter, 3 μm in height, and at 2 μm pitch). For the optimization of VNEAs, embryonic rat cortical neurons or HEK293 cells were cultured on VNEAs for few days, and approximately 50% of electrodes instinctively penetrated the plasma membrane. This caused a potential drop across the membrane, and seal resistance was developed between VNEAs, and the plasma membrane was estimated to be 100–500 MΩ [52,53]. However, without penetration of plasma membrane, scientists also have developed the transient electroporation method by NW to estimate AP [54]. Recently, a complementary metal–oxide–semiconductor (CMOS) electrode array (CNEA) was fabricated to bridge the void between MEAs and patch-clamp arrays. It comprises 1024 recording/stimulation pixels, and each pixel is equipped with a vertical nanoelectrode to estimate intracellular membrane potentials from hundreds of connected in vitro neonatal rat ventricular cardiomyocytes. Moreover, it was suggested that after modification and refinements in the device, it can be used for the cultured neuron and tissue preparations and opens new opportunities for basic studies of electrogenic cells and their network [51]. Furthermore, nanotechnology coupled to microtechnology might also develop a special electrode to record activity. With regard to this, in 2016, Wijdenes et al. designed a neuro-electronic hybrid technology and a planar microelectrode array with nanoedges (between 5 to 15 nm height and 2 to 3 μm width) for high fidelity recording at around 15 times higher resolution than normal planar electrode and long-term signal recording (≥30 days) of cultured neurons [55]. As neuronal cell adhesion and strong contact with the recording site are fundamentals of longer sustainable recording, a conventional 3D electrode cannot be used for longer time recording. Cultured neurons are likely to pull away from the recoding sites because of physical tension created by either elongated neurites or growth cones, which leads to weakened contact with the recording site and thereby reduces the signal recording efficacy and causes neuronal membrane damage [52]. The planer nanoedge microelectrode reserves and maintains contact with neurons by preventing their migration away from electrodes but, at the same time, did not limit the neuron movement caused by physical tension. Thus, neuronal integrity was not compromised, which enabled the neuronal recording for a longer time (at least two weeks) [55].

Even though it has been considerably studied where electric responses by the neuronal cells are induced by applying voltage, less is known about the mechanical response of neuronal cells when excited electrically [56,57]. The role of these mechanical interactions is very important in cell biology and physiology. For instance, in neuronal cells, mechanical processes, such as dendritic and axonal elongations, regulate their synapse formation and structural remodeling [58]. Nguyen et al. (2012) developed a piezoelectric PbZr_x_Ti_1−x_O_3_ (PZT) nanoribbons, which could detect the cell deflection of 1 nm when 120 mV is applied to the cell membrane. The cell line used for the experiment was rat pheochromocytoma (PC12 cells), which is similar to sympathetic neurons when treated with nerve growth factor (NGF). The measured deflections resembled the theoretical model where applied voltage causes cell depolarization and leads to alter membrane tension, which makes the cell change its radius to keep pressure constant across the membrane [57,59,60]. Another study for intracellular recording was conducted by Zhao et al. (2019) using a U-shaped nanowire field-effect transistor (U-NWFET). Similar to patch-clamp, U-NWFETs showed abilities to a multiplexed recording of full amplitude intracellular APs from primary neurons and other electrogenic cells [61]. The discussed research, if implied, could enable future investigations and provide future directions in neurotechnology research.

## 3. Challenges and Future Perspectives

Undoubtedly, nanotechnology has provided numerous benefits over microtechnology for culturing neuronal cells in vitro. Moreover, the fabrication of different nanostructures and their integration with microtechnology have offered in vivo-like environments. However, there are still a few challenges that need to be addressed. The critical challenges found in the aforementioned four key areas are: (i) a lack of fundamental understanding of the mechanisms governing the formation and elimination of synaptic connections for precise neural network, (ii) difficulties in developing the platform where multiple cell lines can be appropriately programmed for controlling cell behavior and morphology, (iii) optimization of the stimulus parameters, such as type of stimulation, stimulation power and duration, avoiding inappropriate stimulations which lead to apoptosis, and (iv) biosafety issues caused by cytotoxicity and genotoxicity of nano-sized materials.

In particular, nanotoxicity has emerged as one of the major concerns with using new nanomaterials for cell culture or in vivo application [62,63,64,65]. As discussed in this review, several nanostructures, such as nanowire, nanotubes, and nanopillars, are being used for their application in neural cell engineering. In recent years, few studies have shown their negative effects; for instance, CNTs can harm the cells by inducing oxidative stress, genetic damage, inflammation, and some long-term pathological effects. Additionally, the translation of this technology to preclinical or clinical applications is often very difficult due to the lack of correlation of NSC behavior between in vitro and in vivo and standardization of nanomaterials [66]. The impact of different nano topographies and patterns on neural cells needs to be studied for constructing neuronal networks and their application in neuroscience. To reduce and eliminate nanotoxicity, it is crucial to understand how nanoparticles interact with living cells and other biological systems. It is necessary to have a thorough understanding of the mechanisms of interaction between nanoparticles and target cells that might have local and systemic effects in preclinical and clinical applications. Other suitable strategies include reduction of toxic composition, reduction in the length of exposure, control of both size and shape of the employed nanomaterials, and adjust surface properties of the nanomaterials by coating them [67].

Furthermore, there is a need to emphasize finding the accurate balance of microenvironment factors to obtain the pure population of differentiated neural cells. The sorting of differentiated populations, due to the absence of control on the differentiation process, may limit their application on larger scales. To overcome this, a specific microenvironment for neural stem cell differentiation should be discovered. Another major concern that needs to be addressed is the complexity of fabricating 3D porous scaffolds with certain nanotopographies on them. Although at a small scale, the development of nanotopographies on the biomaterial has been achieved, and these nanotopographies had shown good interaction with neural cells to establish a neural network. However, nanopatterning technologies for biomaterials on a large scale need to be achieved. Moreover, the biocompatibility and biodegradability of the nanomaterials need to be checked properly before their use in vitro and in vivo. These technologies can provide 3D neuronal cultures, which will not only offer the platforms for toxicology assays but also help in designing future biocompatible devices. Upcoming advancements in this field will develop sophisticated, more functional, and safer platforms for its application in both clinical and scientific endeavors.

## 4. Conclusions

In summary, we have highlighted how nanotechnology can be used to guide neural stem cell growth and their differentiation in in vitro culture. Nanotechnology-based methods can be customized to regulate the neuronal cell culture for establishing an in vivo-like model for research. The scaffold developed using nano/microtechnology for stem cell applications is biomimetic and biocompatible and supports neural cell growth and differentiation. Biomaterials with some specific nanotopography have shown their ability to control cell behavior, morphology, and fate. The interaction of different nanostructures with neuronal cells can be stimulated (electric and laser) for different purposes, such as more proliferation, enhancement of neurite length, and differentiation of neural cells. The development of nanotechnology devices, such as nanoelectrode arrays, with circuit integration technology can be used to estimate intracellular membrane potentials and postsynaptic current from hundreds of connected neurons to form networks in vitro. This would be a potential step to discover the relationships between neuronal circuit connectivity and their pathophysiological functions. Furthermore, the cultured neuronal network can play a pivotal role in finding cures/answers for many neurodegenerative diseases which cannot be solved through simple in vivo cell culture models.

## Abbreviations

CNS: Central Nervous System; NSC: Neural Stem Cells; ECM: Extracellular Matrix; CNT: Carbon Nanotubes; MWCNT: Multi-Walled Carbon Nanotubes; PDMS; Polydimethylsiloxane; SH: Superhydrophobic; ND: Nanodiamond; MARC: Multi-architectural Chip; hESCs: Human Embryonic Stem Cells; NRs: Nanorods; ROS: Reactive Oxygen Species; NPs: Nanoparticles; BNNTs: Boron Nitride Nanotubes; CNFs: Carbon Nanofibers; VNEAs: Vertical Nanowire Electrode Arrays.
